# Does frequent tea consumption provide any benefit to cognitive function in older adults? Evidence from a national survey from China in 2018

**DOI:** 10.3389/fpubh.2023.1269675

**Published:** 2023-11-03

**Authors:** Chen Wei, Jiao Zhang, Na Chen, Zhou Xu, Huang Tang

**Affiliations:** ^1^School of Health Economics and Management, Nanjing University of Chinese Medicine, Nanjing, China; ^2^Center of Health Policy and Management Studies, Nanjing University, Nanjing, China; ^3^School of Elderly Services and Management, Nanjing University of Chinese Medicine, Nanjing, China; ^4^Nanjing Hospital of Traditional Chinese Medicine, Nanjing, China

**Keywords:** tea consumption, cognitive function, older adults, propensity score matching, mediating effect

## Abstract

**Objectives:**

This present study aims to investigate the effect of tea consumption on cognitive function and examine possible psychosocial mechanisms in older adults.

**Participants and methods:**

The data of this study came from the 2018 wave of the Chinese Longitudinal Healthy Longevity Survey(CLHLS), and a total of 11,910 valid samples were included. We used ordinary least squares (OLS) to explore whether frequent tea consumption had significant effect on the cognitive function of older people. The problem of endogeneity was addressed by using a propensity score matching (PSM). Then we further explored the psychosocial mechanisms of the effect using a stepwise regression approach.

**Results:**

Frequent tea consumption produced a positive effect on Mini-Mental State Examination (MMSE) score (coefficient = 0.340, *p* < 0.01), and PSM showed similar results. Specifically, the positive effect of green tea (coefficient 0.409, *p* < 0.01) was significantly greater than the other teas (coefficient 0.261, *p* < 0.1). Moreover, frequent tea drinkers were 59.7, 74.8, and 81.8% less likely to have severe, moderate and mild cognitive impairment respectively, compared to infrequent tea drinkers (*p* < 0.01). Levels of depression and sleep quality had partial mediation effect for frequent tea consumption on cognitive function, accounting for 27.6 and 3.5% of the total effect, respectively.

**Conclusion:**

Frequent tea consumption was found to have beneficial effects on cognitive function, especially in older people with green tea intake. Sleep quality and levels of depression partially mediated the association between frequent tea consumption and cognitive function among Chinese older adults.

## Introduction

Cognitive function refers to the ability of human brain to process, store and extract information, including perception, attention, memory, logical thinking, and other abilities (1). Older adults generally experience decreases in cognitive function with ageing. A recent study revealed that the overall prevalence of dementia is 6.0%, and that of mild cognitive impairment (MCI) is 15.5% among the Chinese older adults, respectively 15.07 million dementia patients and 38.77 million MCI patients (2). Cognitive impairment in older people is associated with a reduced quality of life and an increased risk of death, while it also brings a heavy burden to families and society (3).

Originating in China, tea and tea planting have spread all over the world since the middle of the Tang dynasty. At present, tea has become one of the most widely consumed beverages other than water (4). Biological studies have found that catechins, theanine and other compounds in tea may have neuroprotective effects (5, 6). Tea drinking habit in older people may prevent the decline of memory and associative learning ability by affecting the posterior volume of corpus callosum, contributing to an improvement of the cognitive function (7). In addition, epidemiological investigations from the United States, Japan, Singapore, China and other countries also attempted to reveal the relationship between tea drinking and cognitive function in older people, and most studies concluded that frequent tea consumption was associated with better cognitive function (8–16). However, there are some inconsistent findings. For instances, Xu et al. demonstrated that the benefits of tea drinking were restricted to cognitively healthy older adults but not older adults with MCI (17). Ide et al. reported that green tea consumption over 12 months may not significantly affect cognitive function measured by the Japanese version of the MMSE (18). Drinking green tea, but not black tea or others, was found to be associated significantly with a reduced risk of cognitive decline (19–21).

Other studies have focused on the effects of tea consumption on depression and sleep quality. It has been shown that frequent tea drinkers have significantly lower levels of depression (22–27) and that frequent tea drinking is effective in improving sleep quality in older adults (28–30), while depression and sleep disturbance are often considered risk factors for cognitive function in older people (31–35). Therefore, we hypothesise that depression levels and sleep quality may play mediating roles in the association between frequent tea consumption and cognitive function.

Among the previous studies on the relationship between frequent tea drinking and cognitive function, some of them were randomized controlled trials, the sample sizes of which were often too small to draw strong inference; and others using epidemiological data of large samples were likely to suffer from endogeneity problems such as self- selection bias, which might lead to unreliable results. Based on the data from the 2018 wave of the Chinese Longitudinal Healthy Longevity Survey (CLHLS), this article used ordinary least squares (OLS) to explore whether frequent tea consumption had a significant impact on the cognitive function of older people. We addressed the problem of endogeneity by using a propensity score matching (PSM) approach. In addition, few studies have been conducted to explore the mediating effects of depression and sleep quality on the relationship between tea drinking and cognitive function in older adults. This paper further explored the mechanisms of tea drinking on cognitive function. Therefore, our study fills a gap in the literature and contributes to a more integrated analysis of tea consumption on cognitive function in older people.

## Methods

### Data source

**Figure 1:**
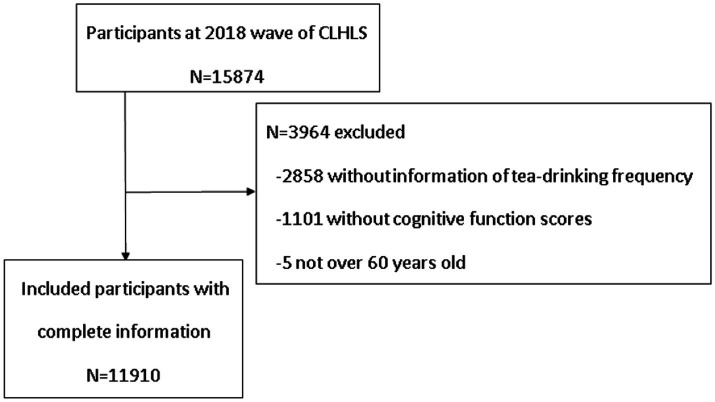
Flowchart of the study population.

The data in this paper came from the 2018 wave of CLHLS, published by the Centre for Healthy Ageing and Development, Peking University. The 2018 CLHLS used a stratified sampling method to investigate the demographic characteristics, dietary habits, behaviors and lifestyles, health status, and other relevant information of older adults people in 23 provinces of China, with a total of 15,874 samples. Excluding samples under 60 years of age, and those with missing data on key variables, a total of 11,910 valid samples were obtained for this study (see Figure 1).

### Variables

#### Dependent variable

CFS (cognitive function score) was the dependent variable in this study. The Mini-Mental State Examination (MMSE) was designed by Folstein (36), and now it is widely used to measure cognitive function and has good applicability in the Chinese population (37). This survey used a validated Chinese version of MMSE scale to assess 6 areas of cognitive function in older people, including orientation (10 points), immediate memory (3 points), attention and calculation (5 points), delayed memory (3 points), verbal ability (8 points), and visual-spatial skill (1 point). The dependent variable was described by MMSE scores, ranging from 0 to 30, with higher scores indicating better cognitive function.

#### Independent variables

In this study, the key independent variable was defined as whether one drank tea frequently, represented by TEA. The participants were asked to report their past frequency of tea drinking at around age 60 as well as their current frequency of tea drinking. If the answer was “almost every day,” the participant was considered to be a frequent tea drinker and the variable tea was assigned a value of 1. If the answer was “at least once a week,” “at least once a month,” “sometimes” or “rarely or never,” the participant was considered to be an infrequent tea drinker and the variable tea was assigned a value of 0.

#### Control variables

Based on previous studies (38–40), three types of variables were treated as control variables in this study. The first type of control variables described the demographic characteristics, which included Gender (1 = male; 0 = female), Age (continuous variable), Marriage (1 = married; 0 = single, divorced or widowed), Resident (1 = living in urban areas; 0 = living in rural areas), Education (years of schooling, continuous variable), and SES (socio-economic status: 1 = good; 0 = poor). The lifestyles were the second type of control variables, including Living arrangement (1 = living with family, 2 = living alone; 3 = living in an institution), Smoke (1 = yes, 0 = no), Drink (drinking alcohol regularly, 1 = yes, 0 = no), Exercise (1 = yes, 0 = no), Examination (regular physical examination once every year, 1 = yes; 0 = no), and Interact (frequency of interacting with friends: 1 = no; 2 = sometimes; 3 = monthly; 4 = weekly; 5 = daily). The third type of control variables were health statuses, including subjective health and objective health. We used a score of SRH (self-rated health: 1 = very bad; 2 = bad; 3 = fair; 4 = good; 5 = very good) to measure the subjective health, and the objective health was measured by a score of ADL (activities of daily living: 1 = strongly limited; 2 = limited; 3 = not limited).

#### Mediating variables

There were two mediating variables in this study, including depression scores and sleep quality. The 10-item Center for Epidemiologic Studies Depression Scale (CESD-10), which has shown good reliability and validity (41), was employed to measure the depressive symptoms. The variable Depression was created as a total score for the 10 items, and each of them was scored 0–3. Higher scores denoted greater severity of depression, with a total score range from 0 to 30. In addition, the variable Sleep was created and assigned a value of “1–5” for the current sleep quality of “very bad, bad, fair, good and very good.”

#### Statistical analyses

As the dependent variable was treated as a continuous variable, this study constructed a multiple regression model to analyze the relationship between tea drinking and cognitive function, and the regression equation is shown below.


(1) $${CFS}_{i}=\alpha_{0}+\alpha_{1}\times {\text{TEA}}_{i}+\alpha_{2}\times {CV}_{i}+\varepsilon_{i}$$

where *i* indicated individual, CFS*_i_* denoted the cognitive function score of older adults, TEA*_i_* stood for whether tea was consumed frequently, CV*_i_* represented the control variables, including demographic characteristics, lifestyles and health statuses, *α*_0_ indicated the intercept term, *α*_1_ was the regression coefficient of variable TEA, which was our main interest, *α*_2_ denoted the coefficients of control variables, and *ε_i_* was the error term.

Furthermore, using this multiple regression model, we examined the robustness of this research by replacing the independent and dependent variables separately. We focused on the effects of drinking different types of tea and different frequencies of tea drinking on cognitive function scores, as well as the effects of frequent tea drinking on cognitive function status.

Propensity score matching (PSM) can address the selection bias caused by observable individual heterogeneity, and bring the observed data close to the randomized trial data. In this study the propensity score, defined as the conditional probability that an individual was a frequent tea drinker based on the observable characteristics, was calculated from the logit regression coefficient. Average treatment effect on the treated (ATT) was estimated as follows:


$$ATT=E\left(Y_{1i}-Y_{0i}|D_{i}=1\right)=E\left(\;Y_{1i}|\;D_{i}=1\right)-E\left(\;Y_{0i}|\;D_{i}=1\right)$$

where $E\left(Y_{1i}|D_{i}=1\right)$ suggests the average MMSE score of the older adults drinking tea frequently, and $\left(\;Y_{0i}|\;D_{i}=1\right)$ is the counterfactual outcome, which indicates what the average MMSE score of the older adults drinking tea frequently would have been if they had not drunk tea frequently.

We attempted to test whether frequent tea consumption could protect cognitive function by reducing depression levels and improving sleep quality using a stepwise regression approach. Eq. (2) was employed to analyze how independent variable TEA affect variable MEDIA. After addition of variable MEDIA, Eq. (3) was used to check whether the effect of tea drinking on the cognitive function still existed and whether it was quantitatively changed. The specification of econometric model is as follows:


(2) $${\text{MEDIA}}_{i}=\beta_{0}+\beta_{1}\times {\text{TEA}}_{i}+\beta_{2}\times {CV}_{i}+\mu_{i}$$

where MEDIA*_i_* denoted mediating variable, which referred to the variable of Depression and Sleep, respectively. TEA*_i_* indicated whether tea was consumed frequently, CV*_i_* represented the control variables, *β*_0_ suggested the intercept term, *β*_1_ was the coefficient of TEA*_i_*, which was our main interest, *β*_2_ stood for the coefficients of control variables, and *μ_i_* was the error term.


(3) $${CFS}_{i}=\gamma_{0}+\gamma_{1}\times {\text{TEA}}_{i}+\gamma_{2}\times {\text{MEDIA}}_{i}+\gamma_{3}\times {CV}_{i}+\sigma_{i}$$

where CFS*_i_* denoted the cognitive function score of older adults, TEA*_i_* indicated whether tea was consumed frequently, MEDIA*_i_* suggested mediating variable, CV*_i_* represented the control variables, *γ*_0_ indicated the intercept term, *γ*_1_, the coefficient of TEA*_i_* and *γ*_2_, the coefficient of MEDIA*_i_* were our main interest, *γ*_3_ indicated the coefficients of control variables, and *σ_i_* was the error term.

In this study, Stata 17.0 (Stata Corp, College Station, TX) was used to conduct descriptive statistics, construct OLS regression and mediating effects models, and perform PSM and robustness test. Moreover, all tests were two-sided, with a *p* value <0.05 indicating statistical significance.

## Results

### Descriptive statistics

Table 1 displays the results of the descriptive statistics. There were 17.20% of the older adults who were frequent tea drinkers in the sample of 11,910, and the mean cognitive function score on MMSE in the full sample was 24.43 ± 6.76. The frequent tea drinkers had a higher MMSE score (26.40 ± 5.35), compared with participants who reported never drinking tea frequently (mean score: 24.02 ± 6.94). ANOVA revealed that the MMSE score was significantly different between the two groups (*p* < 0.001). Among the study population, 44.26% were male, 43.40% were married, and 29.49% were urban residents. We found the mean age of respondents was 84.39 ± 11.67 years, and their mean education was 3.43 years. Additional information is also provided in Table 1, and significant differences in each parameter were observed between the two groups (*p* < 0.001).

**Table 1:** Characteristics of the whole study population and its subgroups by independent variable.

Variable/subgroups	Total sample	Frequent tea-drinking	Infrequent tea-drinking	*p* value
Sample, *n*	11,910	2,048	9,862	
%	100.00	17.20	82.80	
CFS	24.43 ± 6.76	26.40 ± 5.35	24.02 ± 6.94	<0.001
Male (%)	5,271 (44.26)	1,351 (65.97)	3,920 (39.75)	<0.001
Age	84.39 ± 11.67	81.47 ± 11.18	85.00 ± 11.67	<0.001
Married (%)	5,169 (43.40)	1,176 (57.42)	3,993 (40.49)	<0.001
Urban residents (%)	3,512 (29.49)	917 (44.78)	2,595 (26.31)	<0.001
Education	3.43 ± 4.24	5.65 ± 4.81	2.96 ± 3.96	<0.001
SES good (%)	10,260 (86.15)	1,845 (90.09)	8,415 (85.33)	<0.001
Living arrangement	1.23 ± 0.50	1.19 ± 0.47	1.24 ± 0.51	<0.001
Smoker (%)	1,777 (14.92)	519 (25.34)	1,258 (12.76)	<0.001
Alcohol drinker (%)	1,704 (14.31)	496 (24.22)	1,208 (12.25)	<0.001
Regular exercise (%)	3,881 (32.59)	940 (45.90)	2,941 (29.82)	<0.001
Regular physical examination (%)	8,165 (68.56)	1,472 (71.88)	6,693 (67.87)	<0.001
Interacting with friends	2.76 ± 1.71	2.87 ± 1.69	2.74 ± 1.71	<0.001
SRH	3.39 ± 0.95	3.51 ± 0.95	3.37 ± 0.95	<0.001
ADL	2.56 ± 0.67	2.65 ± 0.61	2.54 ± 0.68	<0.001

*p* values are based on chi-square test (*n*, %) or ANOVA (mean ± SD).

### OLS regression

Table 2 shows the OLS regression results of the effect of tea drinking on cognitive function score. According to Model 1, we found that frequent tea consumption produced a significantly positive effect on cognitive function score (coefficient = 2.382, *p* < 0.001), but this model had a goodness-of-fit of *R*^2^ = 0.018, which was not satisfactory. With the addition of demographic characteristics and lifestyles variables to Model 2 and 3 in turn, the regression coefficient decreased to 0.493 and 0.406, however *R*^2^ was increased to 0.311 and 0.339, respectively. When we further added the control variables of health statuses to Model 4, the regression results showed that the coefficient decreased to 0.340, which is significant at 0.01 level. The regression results imply that compared to the older adults who did not drink tea frequently, the frequent tea drinkers had an increased MMSE score by 0.340 after adjusting for the control variables.

**Table 2:** OLS regression results of the effect of tea consumption on cognitive function score.

	Model 1	Model 2	Model 3	Model 4
Tea	2.382*** (0.137)	0.493*** (0.123)	0.406*** (0.121)	0.340** (0.117)
Gender		1.240*** (0.113)	1.140*** (0.121)	1.065*** (0.117)
Age		−0.279*** (0.058)	−0.233*** (0.058)	−0.212*** (0.057)
Marriage		0.078 (0.114)	0.160 (0.123)	0.271* (0.120)
Resident		0.936*** (0.130)	1.090*** (0.131)	1.201*** (0.125)
Education		0.131*** (0.014)	0.117*** (0.140)	0.117*** (0.013)
SES		0.896*** (0.159)	0.682*** (0.156)	−0.002 (0.155)
Living arrangement			0.078 (0.118)	0.178 (0.112)
Smoke			0.112 (0.136)	0.035 (0.133)
Drink			0.071 (0.135)	−0.276* (0.132)
Exercise			0.940*** (0.098)	0.474*** (0.096)
Examination			0.943*** (0.123)	0.806*** (0.118)
Interact			0.535*** (0.030)	0.387*** (0.029)
SRH				0.976*** (0.065)
ADL				1.597*** (0.098)
Constant	24.021*** (0.070)	45.791*** (0.505)	39.588*** (0.576)	31.535*** (0.649)
*R*-squared	0.018	0.311	0.339	0.387

Robust standard errors are given in parentheses; ***p < 0.001, **p < 0.01, and *p < 0.05.

Furthermore, the regression results from Model 4 indicate that being a male (coefficient = 1.065, *p* < 0.001), being married (coefficient = 0.271, *p* < 0.05), living in urban areas (coefficient = 1.201, *p* < 0.001), having more years of education (coefficient = 0.117, *p* < 0.001), exercising regularly (coefficient = 0.474, *p* < 0.001), attending regular physical examination (coefficient = 0.806, *p* < 0.001), Interacting with friends frequently (coefficient = 0.387, *p* < 0.001), as well as having a high score of SRH (coefficient = 0.976, *p* < 0.001), and ADL (coefficient = 1.597, *p* < 0.001) had a significant positive effect on cognitive function in older people. We also obtain evidence indicating that being older (coefficient = −0.212, *p* < 0.001), and drinking alcohol regularly (coefficient = −0.276, *p* < 0.05) had a significantly negative effect on MMSE score.

### Propensity score matching

This study was not a randomized trial, and the participants were not randomly assigned to the intervention group or the control group. Individual characteristics may influence not only cognitive function in older people, but also whether he or she drank tea frequently as well. Therefore, due to self-selection bias, the OLS regression results should be viewed with caution. We next used a propensity score matching (PSM) approach to tackle the endogeneity problem caused by self-selection.

Treating whether an individual drank tea frequently or not as the dependent variable, the binary logit estimations are presented in Table 3. The logit regression results showed that the individual characteristics variables of Gender, Age, Resident, Education, Sex, Living arrangement, Smoke, Drink, and Exercise had a significant effect on whether individuals drank tea frequently.

**Table 3:** Logit regression estimation results.

Tea	Coef.	Std. Err.	*Z*	*P* > |*z*|
Gender	0.612***	0.060	10.14	0.000
Age	−0.008**	0.003	−2.66	0.008
Marriage	0.046	0.067	0.69	0.490
Resident	0.521***	0.062	8.42	0.000
Education	0.068***	0.007	9.99	0.000
SES	0.197*	0.085	2.33	0.020
Living arrangement	0.167**	0.058	−2.89	0.004
Smoke	0.455***	0.068	6.66	0.000
Drink	0.383***	0.068	5.62	0.000
Exercise	0.305***	0.056	5.48	0.000
Examination	−0.033	0.059	−0.56	0.576
Interact	−0.004	0.165	−0.26	0.797
SRH	0.048	0.029	1.67	0.095
ADL	0.031	0.045	0.69	0.491
Constant	−2.169	0.344	−6.30	0.000

***p < 0.001, **p < 0.01, and *p < 0.05.

**Figure 2:**
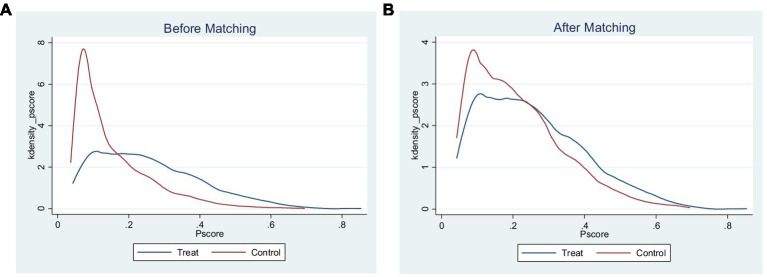
Graphs showed that there was a significant difference in the distribution of propensity scores between the treatment group and the control group before matching **(A)**, while the distributions of propensity scores for the two groups became similar after matching **(B)**.

Then K-nearest neighbor matching, kernel matching and nearest-neighbor matching within caliper of the PSM method were used to conduct a robustness check. Figures 2A,B indicated the distribution of propensity scores before and after matching, respectively. By comparing the distribution of propensity scores before and after matching, it can be visualized that the PSM method significantly corrected the propensity score deviation between the treatment group and the control group.

Table 4 provided the PSM estimation results for the effect of frequent tea consumption on cognitive function. The ATT values from the three matching methods were 0.441, 0.503, and 0.437, respectively. They were all significant at a level of at least 5%, which further demonstrated that frequent tea consumption had a significantly positive effect on cognitive function. After alleviating the endogenous problem of self-selection, the results were generally consistent with the benchmark regression analysis.

**Table 4:** Propensity score matching estimation results.

Method	Treated	Controlled	ATT	S.E.	T-stat
K-nearest neighbor matching	26.397	25.957	0.441*	0.169	2.61
Kernel matching	26.397	25.895	0.503***	0.151	3.33
Nearest-neighbor matching within caliper	26.397	25.960	0.437*	0.168	2.61

***p < 0.001, **p < 0.01, and *p < 0.05.

### Robustness test

In order to ensure the robustness of our conclusions, we conducted a robustness test by replacing the proxy variable of the explained and core explanatory variable. A new explanatory variable Ctea was created to denote the category of tea. We found 9,862 respondents did not drink tea frequently, and the variable Ctea was assigned a value of “0.” Among the 2,048 participants who drank tea frequently, 1,092 respondents drinking green tea were assigned as value “2” of variable Ctea, and 956 samples drinking black tea or other teas was assigned as value “1.” With Ctea as an independent variable. A multiple regression model was constructed by retaining the other variables of Eq. (1). Column Model 1 in Table 5 showed the results that frequent tea consumption was beneficial to cognitive function irrespective of tea category. In contrast however, the positive effect of green tea (coefficient 0.409, *p* < 0.01) was significantly greater than the other teas (coefficient 0.261, *p* < 0.1).

**Table 5:** Robustness test results.

		Model 1	Model 2	Model 3	
		OLS		Multinomial Logit	
		CFS		OCFS	
Ctea	1	0.261* (0.154)			
	2	0.409*** (0.152)			
Ftea	1		0.277 (0.228)		
	2		0.361*** (0.119)		
Tea				1	0.597*** (0.105)
				2	0.748*** (0.077)
				3	0.815*** (0.052)
				4	Base
Control Variables		Yes	Yes	Yes	
Constant		31.544*** (0.650)	31.509*** (0.650)	39.588*** (0.576)	
*R*-squared		0.387	0.388	0.206	

Models 1 and 2 show the regression coefficients of dependent variables, and model 3 shows relative risk ratios of dependent variable. All the robust standard errors are given in parentheses; ***p < 0.01, **p < 0.05, and *p < 0.1.

Another explanatory variable, Ftea, which indicates the frequency of tea drinking, was assigned a value of “0” for rarely or never tea drinking, “2” for drinking tea almost every day, and “1” for drinking tea less frequently in between. Similarly, the results of multiple regression were reported in Column Model 2 of Table 5. Compared to no tea drinking, only frequent tea drinking (coefficient = 0.361, *p* < 0.01) had a positive effect on cognitive function, and low frequency of tea drinking did not have a significant effect on cognitive function.

In addition, the explained variable OCFS was created to measure the ordered cognitive function score instead of the original dependent variable for this study. According to the MMSE scale, a score of 10 or less is considered severe cognitive impairment; 10–20, moderate cognitive impairment; 21–26, mild cognitive impairment; 27–30, normal. From severe cognitive impairment to normal, the older adults of these four subgroups accounted for 5.7, 14.3, 27.4, and 52.6% of the total population respectively, and were assigned a value of “1–4” in turn. Taking OCFS as the dependent variable, multinomial logistic regression analysis was used to calculate the RRR (relative risk ratios). Column Model 3 of Table 5 showed that frequent tea drinkers were significantly 59.7, 74.8, and 81.8% less likely to have severe, moderate and mild cognitive impairment respectively, compared to infrequent tea drinkers (*p* < 0.01). The robustness analysis not only verified the reliability of the results of the benchmark regression, but also provided more detailed information on the causal relationship between tea drinking and cognitive function.

### Mechanism analysis

The above studies revealed the benefits of frequent tea consumption on cognitive function in older adults comprehensively, but a question still remained unanswered: how does frequent tea consumption affect cognitive function? Table 6 displayed the mediating effects of depression and sleep quality on the association between tea drinking and cognitive function.

**Table 6:** Mediating effects of depression and sleep quality.

Variables	(1)	(2)	(3)	(4)	(5)	(6)
	CFS	Depression	CFS	CFS	Sleep	CFS
Tea	0.340** (0.117)	−0.297* (0.122)	0.246* (0.114)	0.340** (0.117)	0.054* (0.025)	0.328** (0.117)
Depression/Sleep			−0.316*** (0.011)			0.231*** (0.055)
Control variables	Yes	Yes	Yes	Yes	Yes	Yes
Constant	31.535*** (0.649)	17.767*** (0.618)	37.142*** (0.650)	31.535*** (0.649)	1.726*** (0.117)	31.135*** (0.656)
*R*-squared	0.387	0.324	0.445	0.387	0.149	0.389
Mediating effects	0.094			0.012		
Mediating effects/Total effects	0.276			0.035		

***p < 0.001, **p < 0.01, and *p < 0.05.

The results of stepwise regressions, taking depression as the mediating variable were summarized in columns (1)–(3) of Table 6, corresponding to Eqs. (1)–(3). As can be seen from column (1) of Table 6, frequent tea consumption positively affected cognitive function (coefficient = 0.340, *p* < 0.01). The regression results in column (2) showed that frequent tea consumption significantly reduced the level of depression in older adults (coefficient = −0.297, *p* < 0.05). Column (3) presented that the coefficient of tea remained significantly positive (coefficient = 0.246, *p* < 0.05), implying a direct effect of frequent tea consumption on cognitive function after controlling for variable depression. Meanwhile, the coefficient of depression was significantly negative (coefficient = −0.316, *p* < 0.001), suggesting that a reduction in depression scores significantly enhances cognitive function. Therefore, depression partially mediated the relationship between tea consumption and cognitive function. The mediating effect accounted for 27.6% of the total effect.

In addition, we used Sleep as a mediating variable to test for mediating effects. As shown in columns (4)–(6) of Table 6, frequent tea consumption significantly improved sleep quality in older adults, which in turn significantly contributed to cognitive function. We captured that sleep quality had partial mediation effect for frequent tea consumption on cognitive function, accounting for 3.5% of the total effect.

## Discussion

In this large population-based study, we observed that older people who frequently consumed any type of tea was associated with a higher MMSE score. This finding was in line with many previous studies. The tea leaves contain various bioactive components that confer multiple health benefits, which may explain the effect of tea on cognitive function. Catechin has been well demonstrated to affect the brain by relieving stress and improving mood (13), and catechins also have been reported to possess antioxidant, anti-inflammatory and neuroprotective effects (14). However, only with a high frequency of tea consumption, these components could reach a certain concentration in the blood to protect cognitive function effectively (5).

Preparation methods can vary greatly among different types of tea. Green tea, which has the greatest market share, is the only non-fermented tea that offers the best protection for cognitive function in older people. Relatively, black tea is a fully fermented tea and the fermentation process usually reduces the content of active ingredients such as catechins and theanine, which makes possible explanations for some studies suggesting that black tea consumption in older people was not associated with cognitive decline (19).

Two studies, from the United States and Japan (18, 19), did not find a significant association between tea consumption and cognitive function scores, while the findings from studies in older adults Chinese or overseas Chinese were highly consistent with our research conclusions (8–12). In China, the “tea ceremony” was formed in the Tang Dynasty or earlier. Tea drinking was not only regarded as a good dietary habit, but also an important social activity and a way to cultivate the body and mind by making, appreciating and tasting tea. Therefore, we suggested that older Chinese people who drank tea frequently were psychologically healthier, and levels of depression played an important mediating role between tea consumption and the cognitive health of older Chinese adults. Besides physiological mechanisms, psychosocial factors may also play a role on the effect, which was a noteworthy aspect observed in this study. However, more research is warranted to shed light on this.

This study explored mediation effect of sleep quality on associations between tea consumption and cognitive function. Actually, the effect of tea consumption on sleep quality is controversial. Tea contains caffeine, which is generally considered to be one of the sleep disturbing factors. However, a recent study suggested that tea active ingredients can enhance sleep quality through neuroendocrine pathway, immune system and intestinal microbiota (30). In our study the overall effect of frequent tea consumption on sleep quality was found to be significantly positive, but the robustness and heterogeneity of this effect needs to be further tested.

This study has practical implications. On the one hand, middle-aged and older adults should be encouraged to cultivate a habit of tea drinking as early as possible, which has some effect on protecting cognitive function. On the other hand, in order to enable healthy ageing, the government ought to guide communities and pension institutions to offer interventions of tea therapy for people at risk of cognitive dysfunction (42, 43).

This present study has made several contributions. To our knowledge, this was the first study that PSM method was conducted to confirm the effects of tea consumption on cognitive function, due to the self-selection bias presented in large-scale epidemiological survey. In addition, by replacing the explanatory and explained variable, robustness testing supplemented a more detailed account of the effects. Finally, this study was the first attempt to reveal the psychosocial mechanisms of the relationship between tea consumption and cognitive health by using mediating effects models.

There are still some methodological limitations in this study. Firstly, all variables were measured through self-report questionnaires, which may be in error with the true values. In addition, data imputation was used to fill in missing values to avoid loss of useful information. The measurement errors above would result in inaccurate quantitative estimation of the effect. Secondly, although a large number of control variables were included in our models, our approaches including PSM, could not rule out the possibility of omitted variables bias. Finally, this study used cross-sectional data so that the causal relationship could be considered less solid. We do not know exactly how long individuals need to continue drinking tea to have an effect on cognitive function.

## Conclusion

In conclusion, we observed beneficial effects of frequent tea consumption on cognitive function, especially in older people with green tea intake. Frequent tea consumption was found to be related with a significantly decreased risk of severe, moderate and mild cognitive impairment. Furthermore, we found that sleep quality and levels of depression partially mediated the association between frequent tea consumption and cognitive function in older adults. Prospective studies or randomized trials in different geographical areas are required to clarify the causality, taking the categories of tea, frequency and duration of tea consumption into account.

## Data availability statement

The original contributions presented in the study are included in the article/Supplementary material, further inquiries can be directed to the corresponding author.

## Ethics statement

The studies involving humans were approved by The Ethical Committee of the Chinese Center for Disease Control and Prevention. The studies were conducted in accordance with the local legislation and institutional requirements. The participants provided their written informed consent to participate in this study.

## Author contributions

CW: Conceptualization, Data curation, Writing – original draft, Writing – review & editing. JZ: Conceptualization, Methodology, Writing – review & editing. NC: Writing – original draft. ZX: Data curation, Writing – review & editing. HT: Conceptualization, Funding acquisition, Writing – review & editing.
